# Minority Influence: An Agenda for Study of Social Change

**DOI:** 10.3389/fpsyg.2022.911654

**Published:** 2022-06-23

**Authors:** Radmila Prislin

**Affiliations:** Department of Psychology, San Diego State University, San Diego, CA, United States

**Keywords:** minority influence, social change, social influence, majority influence, group dynamics

## Abstract

Minority influence research was sparked by Moscovici’s observation about the power of active minorities to instigate social change. This idea invigorated research on social influence, which is evident in a subsequent outburst of studies on minority influence up to the 1990s, followed by a decrease and stabilization in the 2000s and 2010s. In spite of a remarkable scientific output, research on minority influence has not addressed its original question about social change. Rather, it has focused dominantly on the cognitive processes and attitudinal change in response to a minority advocacy or minority mere presence, and, to a lesser degree, to the role of minority influence in decision-making and task groups. To orient research toward social change, a research agenda is presented, along with a few illustrative studies. The proposed agenda focuses on time, interactive (minority ↔ majority), and motivated influence as critical explanatory variables to address in the next phase of research on minority influence in the pursuit of social change.

## Introduction

In the beginning, there was a minority. Those who are non-normative and few originate every social change, seeking to alter the mechanisms within the social structure, including social relations and social organization. It was this insight into the role of minorities that made Moscovici a luminary in social psychology. His book with a telling title “Social influence and social change” (Moscovici, 1976) emphasized the role minorities have historically played in instigating political, scientific, religious, and artistic change. The status quo in these arenas is first broken by the rebellious few. This original momentum may subsequently garner greater support and even turn majorities into advocates for change who, in the process of social cryptomnesia, often forget its originators (Mugny and Pérez, 1989). Yet, a potential majority advocacy for change and, ipso facto, minority advocacy for the status quo (e.g., conservative minorities, see Levine and Kaarbo, 2001) is always an outgrowth of what at the start was a minority idea.

There is likely no better contemporary example of the power of minorities to instigate social change than a growing recognition of the urgent need for the humankind to address the cause and impacts of climate change. Sparked by the early scientific findings about human-induced global warming, the issue was first recognized as an existential threat by only a few whose minority position was often ridiculed, dismissed, distorted, or downplayed. Yet, the minority seems to be prevailing, giving credence to Mahatma Gandhi’s saying that “fist they ignore you, then they laugh at you, then they fight you, then you win.” Though far from the final win, this evolving minority-inspired social change is real and multifaceted, at least in the developed world. It encompasses attitudinal reactions (e.g., growing concerns about climate change), behavioral reactions (e.g., growing use of a clean energy), political impact (e.g., Green parties, which exist in most democratic systems, have entered into several coalition governments, taking premierships in some), and structural changes (e.g., laws mandating elimination of climate-damaging emission by a certain date have been passed in several countries).^1^

Minority origin of change is no less true for research on social influence than any other arena. When first introduced, Moscovici’s idea about the power of minorities to instigate change was very much a minority idea. For decades, research on social influence, which established social psychology as a scientific discipline early in the 20th century, conceptualized influence as a one-way street (Prislin and Crano, 2012). This unidirectional conceptualization postulated influence as flowing exclusively from a majority to a minority. Moscovici broke the spell, pointing out that the conceptualization runs afoul of a self-evident truth about the ever-changing world. His masterful analysis of change in practically every domain of human activity invariably diagnosed minority influence in its origin. Political systems, scientific paradigms, religious dogmas, art movements, and fashion styles are all changed by active minorities who successfully challenge the status quo and offer an alternative (Moscovici and Faucheux, 1972; Moscovici, 1976).

The field took notice. The innovative theorizing about active minorities and their effect on the world revitalized research on social influence. In the subsequent decades, scores of studies have tested (Wood et al., 1994), expanded (Nemeth and Walacher, 1983; De Dreu and West, 2001), and challenged (Latané and Wolf, 1981; Tanford and Penrod, 1984) Moscovici’s ideas. Their generative power is additionally evident in several edited volumes on minorities as *bona fide* influence agents (Butera and Levine, 2009; Martin and Hewstone, 2010; Jetten and Hornsey, 2011; Papastamou et al., 2017). The idea of minority influence is now a defining aspect in all conceptualizations of social influence, researched in parallel with majority influence (for reviews, see Prislin and Wood, 2005; Levine and Prislin, 2013). Indeed, it is part of the social psychology canon as evident by its coverage in nearly all social psychology textbooks, handbooks (e.g., Nemeth, 2012; Butera et al., 2017), and encyclopedias (e.g., Crano, 2010). Also, it has informed efforts to address such important issues as jury deliberations (Nemeth, 1981), minority victimization (Moscovici and Pérez, 2007), terrorism (Chen and Kruglanski, 2009), educational reform (Butera et al., 2021), climate change (Bolderdijk and Jans, 2021), and dietary practices (De Groeve and Rosenfeld, 2022).

**Figure 1:**
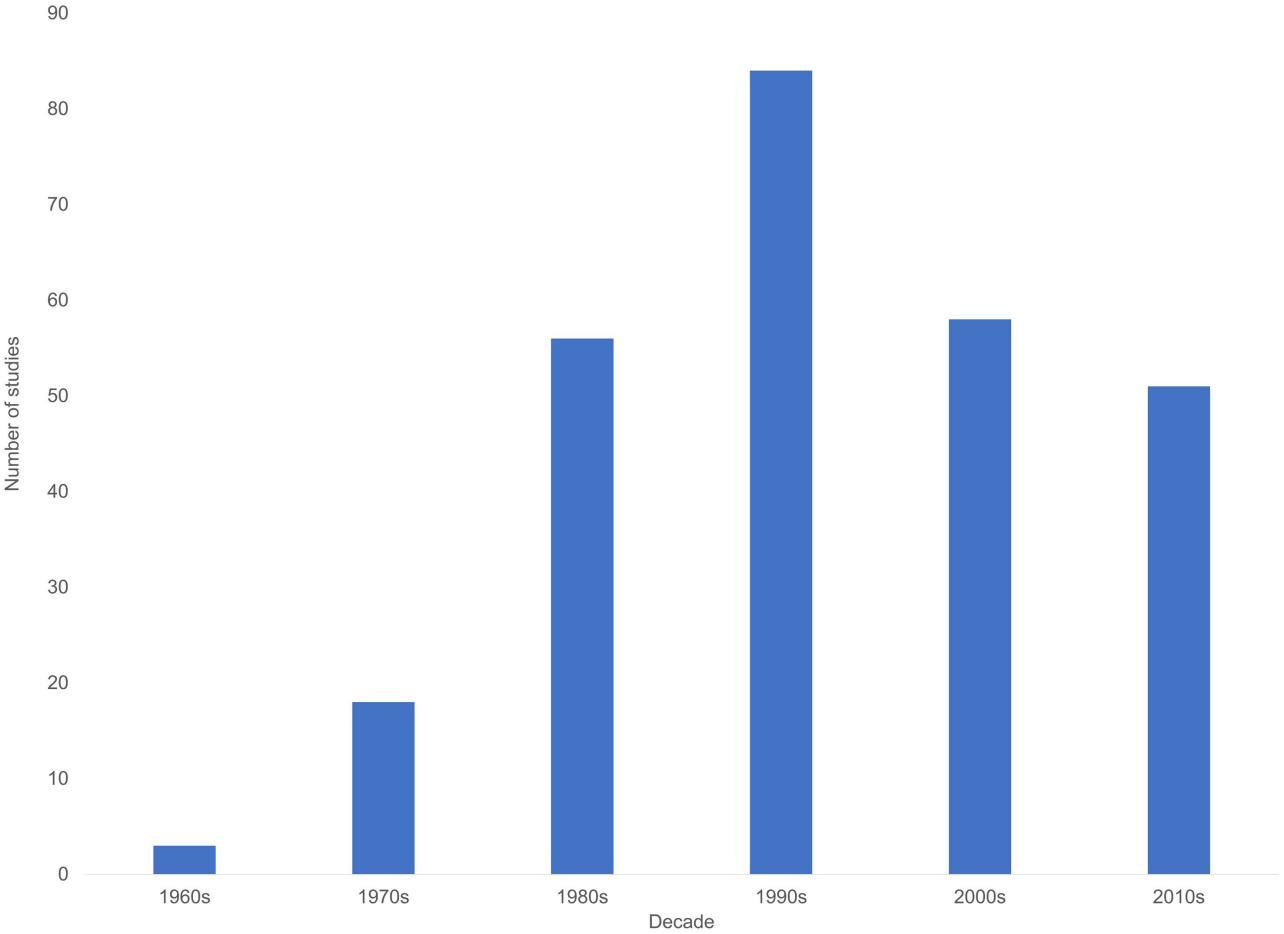
Number of studies on minority influence per decade.

All this testifies to the maturity of research on minority influence. Yet, maturity appears to come at the expense of vigor. Taking the number of published studies as a readily available, however imperfect, indicator of vigor, the findings are telling. An earlier analysis of this index has identified 238 unique empirical studies on minority influence published between 1960, when the foundational research on minority influence first appeared, and December 2011 (see Prislin et al., 2017). An additional unique 33 empirical studies have been published since then and the end of December 2020, as identified searching the PsycInfo database, manually searching outlets in which earlier studies on minority influence appeared, and cross-reference checking.^2^ As evident in Figure 1, after a somewhat slow start—or, one could say, a delayed impact of Moscovici’s ideas—this research saw a steady increase in representation in scientific outlets, reaching its zenith in the 1990s, but gradually declining in the subsequent two decades.^3^

The rise of research on minority influence coincided with the cognitive orientation in social psychology. Reflecting the broader social cognition orientation, this research has focused on cognitive responses to a minority (vs. majority) advocacy. Studies done in this tradition have generated valuable insights into the nature and outcomes of minority influence, specifying conditions under which a minority exerts influence and the processing underlying such influence. Importantly, most of these studies were conducted within the information processing (persuasion) framework, elucidating cognitive responses to a minority (vs. majority) advocacy and eventual attitudinal changes on the objects of advocacy (e.g., Mackie, 1987; Baker and Petty, 1994; Erb and Bohner, 2001; Martin and Hewstone, 2008). As a counterbalance to research on the informational value of minority advocacy, several lines of research have emphasized the role of social identity and the resultant normative pressures in determining reactions to minority influence, which reactions expand beyond attitudinal shifts to include group-categorization (e.g., Abrams and Hogg, 1990; David and Turner, 2001) and self-categorization (e.g., Wood et al., 1996). Several integrative models profitably combine the information processing and social identity approaches, capitalizing on their complementary strengths (Pérez and Mugny, 1996; Crano, 2001). Finally, a separate but still very much cognitive line of inquiry has focused on the styles of thinking inspired by minority (vs. majority) pressures, examining thought modality (e.g., Nemeth, 1986) and complexity (e.g., Gruenfeld, 1995).

For all their valuable insights into an individual’s cognitive responses to a minority advocacy, these approaches are not very informative about the role of minority influence in instigating social change. This state of affairs is rather paradoxical given Moscovici’s original theorizing and ultimately, most minorities’ goals. Being in a minority is a social condition, often characterized by adverse outcomes, tangible and intangible alike (for review, see Prislin and Christensen, 2005a). Minorities have been “pigeonholed, pathologized, stigmatized, and dismissed in a countless way” (Moscovici, 1994, p. 239). It is these social conditions that minorities seek to change.

According to the genetic model of Moscovici (1976), the power of minorities to effect social change lies in their capacity to create a social conflict with majorities through vigorous advocacy of their position. Espousing a behavioral style characterized by temporal consistency, internal consensus, and autonomy and investment in their position, even at the price of reprisals, minorities present a force to be reconed with. While standing firm, minorities must also convey a level of open-mindedness in acknowledging others’ positions to avoid being perceived as rigid (Papastamou and Mugny, 1985). In doing so, minorities make themselves visibly non-normative, hence, creating a social conflict. At the same time, their behavior style invites an attributional analysis that forces a majority to consider the merits of the minority’s non-normative position. This socio-cognitive conflict may be resolved over time such that some majority members privately adopt the minority position. This private conversion may cautiously be communicated to others, thus, gradually becoming public. When a significant number of majority members publicly acknowledge their adoption of the minority position, a new norm is created, effectively turning the initial minority into the majority and vice versa.

Moscovici’s initial theorizing makes it clear that changing an individual’s mind is, at most, a potential intermediatory step toward social change. It cannot be a goal in and of itself. Yet, the prevailing research on minority influence seems to have made it so. The reasons for this development are complex. Likely responding to the disciplinary cognitive Zeitgeist, Moscovici himself replaced his initial rich theorizing about the social underpinnings of minority influence in a pursuit of social change with a theorizing that emphasized cognitive factors. His subsequent conversion theory (Moscovici, 1980) focused exclusively on cognitive processes underlying minority vs. majority influence and the resultant nature of attitudinal (not social!) change. Others followed suit, accumulating findings that cast doubt on the conversion theory postulate about the unique nature of minority (vs. majority) influence processes (for a meta-analytical synthesis, see Wood et al., 1994; see also Erb and Bohner, 2001; Martin and Hewstone, 2008). Moreover, a disciplinary general focus on the individual level of analysis made social change a challenging object of inquiry. Adding to these theoretical and analytical reasons are powerful pragmatic reasons: ever increasing demands on publishing productivity, which is often used as a proxy for scientific contribution, are easier to satisfy with a minimalist approach to minority influence where social conditions and social responses are reduced to information to be processed or generated, with an individual, rather than a group, as a unit of analysis. The latter is simply too costly for a frequent-publication dictum (see Levine and Kaarbo, 2001).

Yet, no amount of normative or pragmatic pressure can extinguish minorities. They exist in research on minority influence, too. Two lines of research: One on group decision-making, and another on the impact that newcomers to groups make, provide alternative approaches to the study of minority influence.^4^ Within these lines of research, minority influence is addressed in the context of interacting groups, with minority and majority positions publicly advocated and hence, memberships in minority and majority factions actively experienced. Research on group-decision making has elucidated the probability of any one faction prevailing as a function of types of decision tasks (Laughlin and Ellis, 1986), and shared cognitions and motivations (Brodbeck et al., 2007; Tindale et al., 2013), especially in the context of jury decision-making (Tindale et al., 2004), and organizational decision-making (De Dreu and West, 2001). An important contribution of research on newcomers to the group is a demonstration that newcomers, who, by definition, are in a minority, may affect the existing members’ overt behavior as a highly relevant but generally neglected variable (Levine and Choi, 2010). This research has also documented many contingencies for such impact, including newcomers’ and the existing group members’ idiosyncratic and shared characteristics (for reviews, see Levine and Prislin, 2013; Levine and Tindale, 2015).

Though developed in parallel without much cross-pollination, research on minority influence within the persuasion framework and the interacting group framework converge to the conclusion that such influence is possible, although under a highly constrained set of circumstances (Levine and Tindale, 2015). These circumstances cannot be easily organized in a unifying model within either the information processing (persuasion) framework (but see Martin and Hewstone, 2008) or the interacting group framework, much less under an omnibus framework. However, as pointed out by Levine and Tindale (2015), the overall findings suggest that in those rare circumstances when minorities do prevail, they tend do so through informational processes, by forcing a majority to question the validity of its position. As for the measures of the minority impact, the two frameworks paint a somewhat different picture: The Information processing (persuasion) framework suggests that the impact is most evident on private responses to indirect measures of attitudes toward issues tangentially related to the object of a minority advocacy (Wood et al., 1994; but see Glaser et al., 2015). In contrast, studies of interacting groups tasked with reaching a consensual decision have frequently demonstrated publicly stated, direct changes in opinions toward focal issues of minority advocacy (Paicheler and Flath, 1988; Smith et al., 1996). This discrepancy suggests that in the sphere of minority influence, as in many other spheres, interacting, mutually interdependent individuals react differently than solitary individuals processing social information. This poses an additional challenge to applying many insights from the prevailing literature on a minority impact toward a social-psychological understanding of social change.

## Minorities Effect Social Change Over Time, Winning a Competition in Influence Exerted for Various Reasons

Even a cursory analysis of just about any social change reveals that it takes time to alter social relations and social structure within a group. The dynamic nature of this process is additionally evident in its competitive nature as minorities and majorities contest each other while advancing their own position. They do so for a variety of reasons, which fuel their influence efforts and shape their influence strategies. To understand social change, it is necessary to study influence efforts as they occur over time, in dynamic exchanges between group factions, which seek to influence for various reasons. Hence, the proposed agenda focuses on time, interactive, and motivated influence as critical explanatory variables to address in the next phase of research on minority influence in the pursuit of social change.

In what follows, the importance of these factors is discussed and illustrated by a few initial explorations. They have been conducted within the framework of the gain-loss asymmetry model of change in minority and majority positions (Prislin and Christensen, 2005a). The model postulates that the many comparative advantages of the majority position over the minority position generally result in the former being preferred over the later. Hence, changes away from the preferred majority position presumably are experienced as losses, with changes away from the minority position presumably experienced as gains. Because gains are experienced less intensely than the comparable losses (Kahneman and Tversky, 1979), changes away from the minority position should elicit positive reactions that are weaker than negative reactions to changes away from the majority positions. Thus, immediately in the aftermath of social change whereby minorities become majorities and vice versa (minority ↔ majority), the former should react only mildly positively, and the latter should react intensely negatively, toward the group in which they switched positions. As a result, the group should be weakened in the immediate aftermath of social change. Over time, however, successful minority’s initial tepid reactions should increase in positivity if their newly won majority position stabilizes within the group (Hypothesis 1, see below Agenda item 1).

Minority’s success at effecting social change and ipso facto, majority’s failure to prevent it, should influence not only their reactions toward the group but also their capacity to further argue their positions. Because of the vital role social support plays in sustaining any argued position (Prislin and Wood, 2005), gaining social support by virtue of social change (minority → majority) should increase a successful minority’s capacity to continue to advocate their position. In contrast, losing social support by virtue of social change (majority → minority) should adversely affect a failed majority’s capacity to further advocate their position. In other words, the outcomes of social influence attempts aimed at effecting versus preventing social change should have reciprocal effects on the sources of social influence (Hypothesis 2, see below Agenda item 2).

Whereas most minorities seek social change, they do so for a variety of reasons that originate from a variety of disadvantageous social, economic, and psychological conditions. The many reasons behind minorities’ pursuit of social change could be broadly recognized as seeking social validation, social control, or social acceptance. The former two motives can be satisfied by recruiting converts to the minority position. Successful conversion of a sufficient number of group members to the minority position reverses positions (majority ↔ minority), representing a loss for the former majority and a gain for the former minority. Because of the earlier discussed asymmetry in reactions to losses and gains, the presumed intensely negative reactions of the former majorities, but only mildly positive reactions of the former minorities, should render the group weak immediately after experiencing social change *via* conversion. On the other hand, minorities motivated to be socially accepted need not necessarily seek converts to their position.^5^ Rather, they may pursue social change by advocating for tolerance. When successful, these minorities redefine what is acceptable within group’s boundaries. The newly established more inclusive group standard encompasses both minorities and majorities as constitutive elements of the group, representing a gain for the former at no cost for the latter. The resultant increase, however mild, in positive reactions among minorities and continuing positive reactions among majorities should render the group changed *via* tolerance stronger than its counterpart changed *via* conversion (Hypothesis 3, see Agenda item 3).

These hypotheses were tested in a program of research whose multiple studies employed the following procedure: Each study included an experimental creation of opinion-based minority and majority factions within a group. These were created by having a naïve participant interact with a number of confederates, each of whom was trained to provide scripted responses during the course of the group’s interaction. Specifically, a minority faction was created by having most confederates oppose the participant’s stance on one or more socially important issues; conversely, a majority faction was created by having most confederates support the participant’s stances. These initially created positions either remained stable throughout the group’s interactions or were reversed when, halfway through the group’s interactions, several opponents (supporters) switched their alliances to support (oppose) the participant. An important feature of this procedure is that participants actively experienced their social position within the interacting group.

### Agenda Item 1: Time Is of Essence

Length of time it takes to effect social change may vary but social change is always a process of evolution. Even a revolutionary change progresses to its culminating tipping point after a period of build-up that requires time. Yet, most studies on social influence, including minority influence, are one-time affairs that examine the effects of single appeals or single group encounters at a discrete point in time. Similarly, reactions to social influence are typically assessed once at a single point in time. At best, and even that rarely, they are assessed twice, with a second assessment intended to detect the hypothesized delayed effect of minority influence on a focal point of its advocacy (David and Turner, 1996; Crano and Chen, 1998, Study 1; 1999).

The reasons for a paucity of longitudinal studies are many. They map on the reasons discussed earlier for strapping minority influence research in the Procrustean bed of the cognitive processing approach, which, along with serious logistic challenges, effectively eliminated time as a variable in this line of research. This state of affairs is unfortunate as time variable is of essence in understanding how minorities go about effecting social change. Without considering time, some questions are never asked and important phenomena are poorly understood. For example, at what point in time do minorities voice their non-normative opinions? How do they shape their influence strategies over time? At what point in time do their efforts begin to effect the presumed private change? How is that private change communicated over time? At what point in time does it become public and sufficiently substantial to change social relations and social structure within a group? These are but some of the important questions that await answers.

As an illustration of the epistemic value of the “time perspective” approach to understanding the consequences of successful minority influence, and a test of the above-mentioned Hypothesis 1, consider a longitudinal study that examined reactions of a minority faction within a group who successfully converted a sufficient number of majority members to its position to effectively turn themselves into a new majority and ipso facto, the former majority into a new minority (Prislin and Christensen, 2005b, Study 2). The effects of this social change on the new majority’s reactions toward the group in which they prevailed were assessed immediately after the change, and one-, two-, three-, four- and five-weeks after the change. In the immediate aftermath of social change, the new majority showed little attachment to the group—a finding consistent with previous studies (Prislin et al., 2000, 2002; Prislin and Christensen, 2002, 2005b). Although they effectively changed their numerical position, the new majority phenomenologically reacted as if they still had been in a minority, reluctant to “make a *salto mortale* into the unknown” of the new position (Moscovici, October 31 2008, personal correspondence). The unknown of the new position, it was hypothesized, was largely due to the seemingly contradictory meaning of the conversion or former opponents’ sway to the minority position that underlies social change. Whereas such conversion is the condition *sine qua non* for a minority to become a majority, it also signals unreliability and ultimately, instability of the majority position. If so, then prolonged experience in the new majority position should be reassuring, facilitating the new majority’s acceptance of the group as their own. Indeed, the results revealed a significant linear increase in positive reactions toward the group over time. The longer the new majority spent secure in its new position, the more similar they perceived themselves to the group and the more attractive they found the group. These findings document how social constructions of influence attempts and group memberships evolve over time, as do reactions to subsequent changes. Only by incorporating a time perspective in our research can these variations be captured (Arrow et al., 2000).

### Agenda Item 2: Influence Is a Dynamic Competitive Game

The dynamic nature of social influence is additionally evident in its multidirectionality, with multiple factions within a group competing to prevail. In the process, they influence and are influenced, adjust their influence strategies and responses, form and dissolve alliances. Social influence is the perpetual competition that shapes social relations by targeting social attitudes. As nothing stands still, any group’s position is only temporary. This dynamic aspect of social influence was recognized in the early theorizing of Moscovici (1976), as well as in the subsequent consideration of minority influence in the intergroup context by Mugny (1982) that includes the power agent (typically normative majority) and the population (typically the target of both minority and majority influence).

The daunting complexity of the social influence dynamics is challenging to capture in a formal model, much less empirical research. The challenge may be great but not insurmountable. It has been addressed by restricting a number of factors that presumably shape the dynamics of social influence and employing mathematical simulations to test proposed models. For example, the dynamic social impact model posits the strength, immediacy, and the number of sources of influence as three critical factors (Latané, 1996). Their simultaneous activation in simulated interactions ultimately yields a system settled into a pattern of overall convergence in social attitudes but importantly, with some clustering of factions holding minority positions (Latané and Nowak, 1997). More recently, social change has been examined as a function of two factors: Indirect minority influence, defined as attitudinal change on culturally relevant issues different from but related to the focal issue of minority advocacy, and cultural drift, defined as mistakes in copying culturally relevant attributes due to small errors in the learning and memory processes. Computational modeling and simulations suggest that indirect minority influence yields gradual social change and diversity, whereas cultural drift generates rapid social change and polarization in a society (Jung et al., 2021). None of the proposed models, however, has been tested empirically outside of computational simulations.

A rare empirical study of the dynamic nature of social influence focused on reciprocity of influence. In a test of the above-mentioned hypothesis 2, variations in minority and majority sources’ persuasibility were examined as a function of their targets’ responsiveness to their influence appeals (Prislin et al., 2011). Minority sources’ persuasibility increased over time when their targets’ increasingly favorable reactions turned them into a majority (minority → majority). Apparently, a hard-earned social capital in the form of former opponents’ support paid high dividends as successful minorities became increasingly efficient in their advocacy. Whereas turning opponents to supporters appeared to make successful minorities “smarter,” successfully retaining initially won support did not affect successful majority’s persuasibility. It remained consistently high over time. In contrast to these sources, those who were consistently rejected to remain in a minority, as well as those who lost social support to be turned from a majority to a minority (majority →minority), lost much of their persuasive efficacy over time. In what appeared a vicious circle of influence failures, these sources became decreasingly convincing in their advocacy with increasingly negative feedback they received. These patterns of socially regulated persuasiveness were documented by coding video-taped sources’ verbal advocacy (Study 1) and by coding their written advocacy as well as an independent audience’s reactions to their written advocacy (Study 2). Consistency in these findings about the reciprocity of social influence suggests that majorities are able to influence not only because they have social support as an argument, as it is axiomatically discussed in the social influence literature, but also because social support enables them to generate convincing arguments. This may be especially true for new majorities (former minorities) who earn their position through social change.

### Agenda Item 3: Minorities Universally Originate Social Change but for Different Reasons

As stated earlier, every social change is initially a minority’s idea.^6^ To understand motives behind minorities’ pursuit of social change, it is useful to compare their position to that of their majority counterparts. Comparative analyses from multiple lines of research have documented an asymmetry in distribution of tangible benefits (e.g., jobs, wealth) and intangible benefits (e.g., validation, status) favoring majorities over minorities. Similar asymmetry but in the opposite direction has been documented in distribution of social burdens (e.g., illnesses, crime), which are heavier on minorities than majorities. The many advantages associated with majority positions, along with the many disadvantages associated with minority positions (for reviews, see Prislin and Wood, 2005; Prislin and Christensen, 2005a), fuel minorities’ attempts at social change.

The complexity of these asymmetries suggests multiple specific motives behind minorities’ pursuit of social change. This has been recognized in the literature on social movements or social actions as vehicles for effecting social change. Although these actions may include members of both minority groups who are typically disadvantaged and members of majority groups who are typically advantaged, it is almost invariably the former who initiate social actions. Understanding their motivations is in the core of psychological approaches to social action, with different models emphasizing a variety of motives that reflect group’s moral, fairness, and identity standards. When accompanied with a sense of group efficacy, they fuel social actions aimed as social change that will improve minority (disadvantaged) groups’ conditions (for review, see van Zomeren, 2015, see also Klandermans, 1997).

Whereas any classification of motives is unavoidably imperfect, three broad motives are readily identifiable from the previously mentioned analysis of minority and majority positions (Prislin and Christensen, 2005a; Prislin and Wood, 2005): Social validation or a sense of correctness, social acceptance or a sense of belonging, and social control or access to tangible benefits (e.g., resources, power). Regarding the social validation motive, minorities, of course, must have an initial sense of correctness in order to start exerting influence (Prislin and Christensen, 2009). Yet, for their initial sense of correctness to survive, minorities must seek broader social support. Such support transforms what initially is typically construed as a minority’s subjective proposition into an “objectively” correct position synonymous with reality (Hardin and Higgins, 1996; Echterhoff and Higgins, 2017). Similarly, although minorities may partially satisfy their motive for social acceptance within their faction, to be fully integrated within a group, they must seek broader acceptance. Such broader acceptance presumably has survival value (Caporael and Baron, 1997). Whereas these two motives for exerting social influence fit the classical dual-motive scheme for responding to social influence (Deutsch and Gerard, 1955), the third, social control motive has been largely neglected in the social influence literature. Yet, even a cursory analysis of “real life” groups will show that this motive features prominently in social influence exchanges.

Adopting a motivational approach holds promise of advancing our understanding of the roles minorities play in originating social change. Social influence exerted by minorities motivated by social control may differ from social influence exerted by their validation-motivated and acceptance-motivated counterparts. For example, in their analysis of minority influence in political decision-making groups, Levine and Kaarbo (2001) argued that influence strategies employed by political minorities may go beyond informational influence to include the reinforcement (i.e., reward, punishment) and procedural strategies (see also Smith and Diven, 2002). The latter strategies are especially likely in the cases of mutual interdependence between majorities and minorities. This is not to say that any single influence strategy is associated with any particular single motive; however, the same strategy may be employed differently in service of different motives. For example, the exhaustively studied informational influence strategy has been limited to message-based appeals whose goal is to change understanding of an issue through evaluation of message content. Yet, the informational strategy could be used differently to emphasize common group identity (social acceptance) or *quid pro quo* (social control) by acceptance-motivated and control-motivated minorities, respectively. Moreover, the informational influence strategy may be combined differently with other strategies (e.g., procedural tools, threats, coalition-building) in service of a particular motive.

Finally, different motives may call for different types of social change. For example, minorities motivated to be socially validated and those motivated to gain social control are likely to pursue social change seeking converts to their position to transform themselves to a majority (minority → majority). This path to social change alters positions within a group but preserves the notion of a dominant position within the group. However, for some minorities (e.g., LGBT, ethnic, racial minorities), this path to social change is neither desirable nor feasible. These minorities, often primarily motivated to broaden their social acceptance, are likely to seek social change advocating for tolerance. Conversion and tolerance are fundamentally distinct paths to social change with different consequences for group dynamics (Prislin et al., 2018). Change *via* conversion tends to weaken a group, at least immediately in its aftermath, as evident in former majority’s (majority → minority) dramatic decrease in group attachment and loyalty but former minority’s (minority → majority) only mild increase in attachment and loyalty to the group. In contrast, and in support of hypothesis 3, change *via* increased tolerance for diversity within a group dramatically increased attachment and loyalty to the group among those former in the minority (minority → majority) while preserving attachment and loyalty to the group among those former in the majority (majority → minority) (Prislin and Filson, 2009; Shafer and Prislin, 2011) In short, recognizing that minority influence originates from a variety of motives expands the scope of influence strategies and types of social changes to be considered.

## Coda

Research on minority influence, which revitalized the field of social influence, is in need of revitalization itself. Turning, or rather, returning to the original observation that the raison d’être for minority influence is social change points to a promising direction. Minorities effect social change over time, winning a competition in social influence, which is exerted in service of various motives. The proposed agenda for future research on social change effected through minority influence is hardly comprehensive. Whereas numerous other factors may be proposed, they likely covary with the included factors of time, competitive influence dynamics, and motivation. Refining the agenda as further theoretical developments and empirical findings outline should be relatively easy. Overcoming the obstacles that moved minority influence research away from elucidating social change is a greater challenge. If there are reasons for optimism, they include signs of an increasing theoretical and methodological diversity in our discipline. Which leaves us with the persistent “publish or perish” culture that discourages high-stakes, time-consuming, logistically demanding, group-oriented research, especially if it is longitudinal. Yet, answering some questions, including those about minority influence in generating social change, requires just such kind of research. As we are currently re-evaluating our standards of conduct in light of repeated replication failures, which some label a “crisis,” it may be an opportune time to re-evaluate the practice of taking publication productivity as a proxy for scientific contribution. One should never waste a good crisis.

## Author Contributions

The author confirms being the sole contributor of this work and has approved it for publication.

## Conflict of Interest

The author declares that the research was conducted in the absence of any commercial or financial relationships that could be construed as a potential conflict of interest.

## Publisher’s Note

All claims expressed in this article are solely those of the authors and do not necessarily represent those of their affiliated organizations, or those of the publisher, the editors and the reviewers. Any product that may be evaluated in this article, or claim that may be made by its manufacturer, is not guaranteed or endorsed by the publisher.

## Footnotes

^1^Examples of minority-instigated social changes, and the literature as a whole, tend to romanticize minority influence (Hewstone and Martin, 2010). Lest we succumb to the same trap, it is worth remembering that every social change, including those that turned disastrous for the world (e.g., Nazi movement), was originated by a minority.

^2^Search was performed using the following key words: minority influence, defiance, deviance, innovation, social influence and change/effect/impact. Selection criteria included: participants’ exposure to a minority dissent with or without an appeal or reasoning for the dissent, which dissent placed the minority at odds with the participants, and report of a measure of influence. To ensure that only unique empirical studies were counted, care was taken not to double-count studies that were published in journals and later summarized in book chapters or vice versa.

^3^A complete list of publications on minority influence referenced here is available from the author upon request.

^4^Minority label used here in no way diminishes the significance and contribution of these two lines of research. Rather, it signifies their under-representation in the “mainstream” (i.e., information-processing) considerations of minority influence. In these lines of research, group-level outcomes (e.g., consensual decisions, problem solving) are of focal interest, with minority influence being one of the multitude of processes underlying such outcomes. Also, the author’s own line of research on group dynamics in the aftermath of social change could be added to this “minority” category in that its focus on group dynamics is underrepresented in minority influence research (Prislin, 2010; Prislin et al., 2017).

^5^Social influence research has traditionally assumed that a motive for social acceptance underlies yielding to social influence (Deutsch and Gerard, 1955), not exerting social influence. To the extent that acceptance means a state of cognitive merging with a group (Baumeister and Leary, 1995), it cannot be satisfied through acquiescence, which implies affiliation, not integration, with others. Integration or acceptance presumes integration with others into the same category, not giving in to others to join their category (Brewer, 1991).

^6^Whereas every social change originates from minorities, it does not mean that every minority invariably seeks social change. One obvious example are elites, though, it could be argued that they represent numerical minorities but not necessarily non-normative minorities. Elites’ efforts at influence are likely to be directed toward preserving, not changing, the status quo. But there also are examples of numerical, non-normative minorities who, while resisting influence from the majority, do not seek social change (e.g., Mennonites in several countries in North and South America). Minorities who accept rather than challenge the status quo, so long as they themselves are not challenged, are a minority within the minority world. Their influence raises important questions about the conditions under which minorities will (non) seek social change.
